# Depressive symptoms and gender differences in older adults in Hong Kong during the COVID-19 pandemic: a network analysis approach

**DOI:** 10.7150/ijbs.69460

**Published:** 2022-06-06

**Authors:** Yu Jin, He-Li Sun, Simon Ching Lam, Zhaohui Su, Brian J. Hall, Teris Cheung, Ming-Zhao Qin, Chee H. Ng, Yu-Tao Xiang, the International Research Collaboration on COVID-19

**Affiliations:** 1 College of Education for the Future, Beijing Normal University, China.; 2 Unit of Psychiatry, Department of Public Health and Medicinal Administration, & Institute of Translational Medicine, Faculty of Health Sciences, University of Macau, Macao SAR, China.; 3 Institute of Advanced Studies in Humanities and Social Sciences, University of Macau, Macao SAR, China.; 4 Centre for Cognitive and Brain Sciences, University of Macau, Macao SAR, China.; 5 School of Nursing, Tung Wah College, Hong Kong SAR, China.; 6 School of Public Health, Southeast University, Nanjing, China.; 7 Center for Global Health Equity, New York University (Shanghai), Shanghai, China.; 8 School of Global Public Health, New York University, NY, USA.; 9 School of Nursing, The Hong Kong Polytechnic University, Hong Kong SAR, China.; 10 Department of Geriatric Medicine, Beijing Tongren Hospital, Capital Medical University, Beijing, China.; 11 Department of Psychiatry, The Melbourne Clinic and St Vincent's Hospital, University of Melbourne, Richmond, Victoria, Australia.

**Keywords:** older adults, depression, network analysis, COVID-19, Hong Kong

## Abstract

**Background:** The 2019 novel coronavirus disease (COVID-19) outbreak had a detrimental impact on the mental health of older adults. This study evaluated the central symptoms and their associations in the network of depressive symptoms and compared the network structure differences between male and female older adults in Hong Kong.

**Methods:** Altogether, 3,946 older adults participated in this study. We evaluated the centrality indicators for network robustness using stability and accuracy tests, and examined the potential differences between the structure and connectivity of depression networks in male and female older adults.

**Results:** The overall prevalence of depressive symptoms was 43.7% (95% CI=40.6-46.7%) in males, and 54.8% (95% CI=53.1-56.5%) in females (P<0.05). Sad Mood, Guilt, Motor problems and Lack of Energy were influential symptoms in the network model. Gender differences were found in the network global strength, especially in the following edges: Sad Mood--Guilt, Concentration--Guilt, Anhedonia--Motor, Lack of Energy--Suicide, Appetite--Suicide and Concentration--Suicide.

**Conclusions:** Central symptoms in the depressive symptom network among male and female older adults may be prioritized in the treatment and prevention of depression during the pandemic.

## Introduction

Mental health problems are common in older adults and associated with poor quality of life, impaired social functioning, and increased risk of chronic medical conditions 1. Since the outbreak of 2019 novel coronavirus disease (COVID-19), the Hong Kong Government has adopted a variety of public health preventive measures, such as the suspension of public transport, the closure of primary and secondary schools, and a total shutdown of non-essential recreational activities (e.g., gymnasium, communal parks, sports facilities, entertainment venues) to mitigate the risks and impacts of the pandemic 2. All these contingent measures and the ever-changing media reports on COVID-19 may increase the risk of mental health problems, worsen existing psychiatric symptoms, and heighten functional and cognitive impairments in older adults 3. Previous studies have found that public health emergencies such as the Ebola outbreak, earthquakes, and severe acute respiratory syndrome (SARS), could trigger mental health issues 4-6. The high prevalence of psychological morbidities has been documented among older adults who are directly or vicariously exposed to life-threatening situations 7. Therefore, it is essential to pay special attention and provide timely interventions to this vulnerable subpopulation.

The Patient Health Questionnaire-9 (PHQ-9) is a self-report measure of depression consisting of nine items matching the Diagnostic and Statistical Manual of Mental Disorders, Fourth and Fifth Editions (DSM-IV, and -V) criteria of Major Depression 8-11. However, depressive symptoms in older adults are heterogeneous in clinical presentation, often resulting in a highly variable manifestation of symptoms across time, especially during the COVID-19 pandemic 3, 12, 13. Hence, it is crucial to determine the most important symptoms and the inter-relationships between depressive symptoms in older adults during the pandemic. The 'most central' symptoms can be potential treatment targets for interventions to address depression among older adults.

One of the approaches to analyzing relationships between disease symptoms is network analysis 14, an advanced framework that can also explain the onset and maintenance of psychiatric disorders 15, 16. This approach assumes that psychiatric problems consist of co-occurring symptoms and their tendencies causally reinforce one another 17, 18. Network analytic tools can tease out the inter-relationships between psychiatric symptoms from clinical data in a symptom network 16, 19, where each symptom is a node and may have strong associations with one another, and relate significantly to other symptoms in the network. Specific links between two symptoms are defined as edges, which represent the strength of the relationship between symptoms 20. Node centrality index (i.e., strength, expected influence, closeness and betweenness) represents the connectedness of a given variable with all other variables in the network 21, 22. Network analyses could examine the inter-relationships among different symptoms and their relative contributions to the model 23. For example, in a network study conducted in European older adults, central depressive symptoms in the depressive symptom network included suicidal thoughts, depressed mood, anhedonia and pessimism 24, which might activate or perpetuate the remaining depressive symptoms. In addition, there were gender differences in the network structure. In another study on the network structure of major depressive disorder (MDD) in European older adults 25, central depressive symptoms included depressed moods, sleep difficulties, guilt and loss of interest or pleasure had fundamental roles in the network model. Further, several studies explored the network structure of depressive symptoms among other populations, such as adolescents 26, 27, college students 28, patients with other psychiatric disorders 29-31, patients with cancer and the general population 32-34.

However, findings from studies of depressive networks in other populations are not directly applicable to older adults during a pandemic, as late-life depression is often accompanied by distinctive factors, such as chronic diseases, bereavement, retirement and poor social support 12, 24, 25. More importantly, the COVID-19 pandemic may trigger similar fear related to the 2003 SARS epidemic in Hong Kong, which killed nearly 300 people in the city 2. In addition, epidemiological data and clinical features differed greatly between male and female older adults 35, 36, but to date, under the depression network model, the gender differences of Hong Kong older adults during the pandemic have not been examined.

Using the network analysis approach, this study evaluated the structure and gender differences of depressive symptoms (depression hereafter) among older adults in Hong Kong during the COVID-19 pandemic.

## Methods

### Setting and participants

This study was carried out in Hong Kong using convenience sampling in March-April 2020. The inclusion criteria were: (1) aged 50 years and above; (2) Hong Kong residents; (3) able to read and understand Chinese; (4) capable of providing electronic written consent. The data were collected with Qualtrics and Google forms based on Facebook, WeChat, and WhatsApp. The Institutional Review Board of Hong Kong Polytechnic University approved the study protocol.

### Measurement

Depressive symptoms were assessed with the PHQ-9 8. The PHQ-9 was developed based on the 9 depressive symptom criteria in the DSM-IV. The nine items of the PHQ-9 include (1) Anhedonia (“Little interest or pleasure in doing things”), (2) Sad Mood (“Feeling down, depressed or hopeless”), (3) Sleep (“Trouble falling asleep, staying asleep, or sleeping too much”), (4) Lack of Energy (“Feeling tired or having little energy”), (5) Appetite (“Poor appetite or overeating”), (6) Guilt (“Feeling bad about yourself - or that you're a failure or have let yourself or your family down”), (7) Concentration (“Trouble concentrating on things, such as reading the newspaper or watching television”), (8) Motor (“Moving or speaking so slowly that other people could have noticed; or, the opposite - being so fidgety or restless that you have been moving around a lot more than usual”), and (9) Suicidal ideation (“Thoughts that you would be better off dead or of hurting yourself in some way”), with each item rated from 0 to 3, and a total score ranging from 0 to 27. A PHQ-9 total score of ≥5 was categorized as “having depression”, and ≥10 was considered as “having moderate to severe depression” 8.

### Network estimation

The informativeness of each item was evaluated using the means of standard deviation (*M_SD_*) 28 and possible items redundancy was checked. The PHQ-9 item values of 1, 2, and 3 were converted to “presence”, while a value of 0 was labelled as “absence” of depressive symptoms.

The Ising model was used based on binary data 16, 37. The enhanced least absolute shrinkage and selection operator (eLASSO) was adopted to regularize the models 38. Model selection was made using the extended Bayesian Information Criterion (EBIC) 39, 40. Each individual depressive symptom comprised nodes, and the relationships between them were edges in the model 41. The network model was fitted with the R-package IsingFit version 0.3.1 16.

To assess the importance of symptoms in the network, we calculated the centrality indices such as excepted influence (EI), strength, betweenness, and closeness. The accuracy and stability of the model were used to examine the robustness of the results with the correlation stability coefficient (CS-C).

### Gender comparison of network characteristics

As recommended previously 24-26, 28, we investigated whether network characteristics differed between both genders. The Network Comparison Test (NCT) compared networks between male and female participants 42.

## Results

Altogether, 4,464 older adults were evaluated, of whom, 3,946 participated in this study with 923 (23.4%) males and 3,023 (76.6%) females. The overall prevalence of depression (PHQ-9 total score ≥ 5) was 52.3% [95% confidence interval (CI)=50.85-53.80%)], while the prevalence of moderate-severe depression (PHQ-9 total score ≥10) was 22.2% (95% CI=21.03-23.49%). Specifically, the overall prevalence of depression was 43.7% (95% CI=40.6-46.7%) in males, and 54.8% (95% CI=53.1-56.5%) in females (P<0.05). The mean age of the participants was 56.63 [standard deviation (SD)=5.73] years and the mean PHQ-9 score was 6.03 (SD=5.47). The demographic characteristics are shown in Table 1. The PHQ-9 item scores are presented in Table S1.

**Figure 1:**
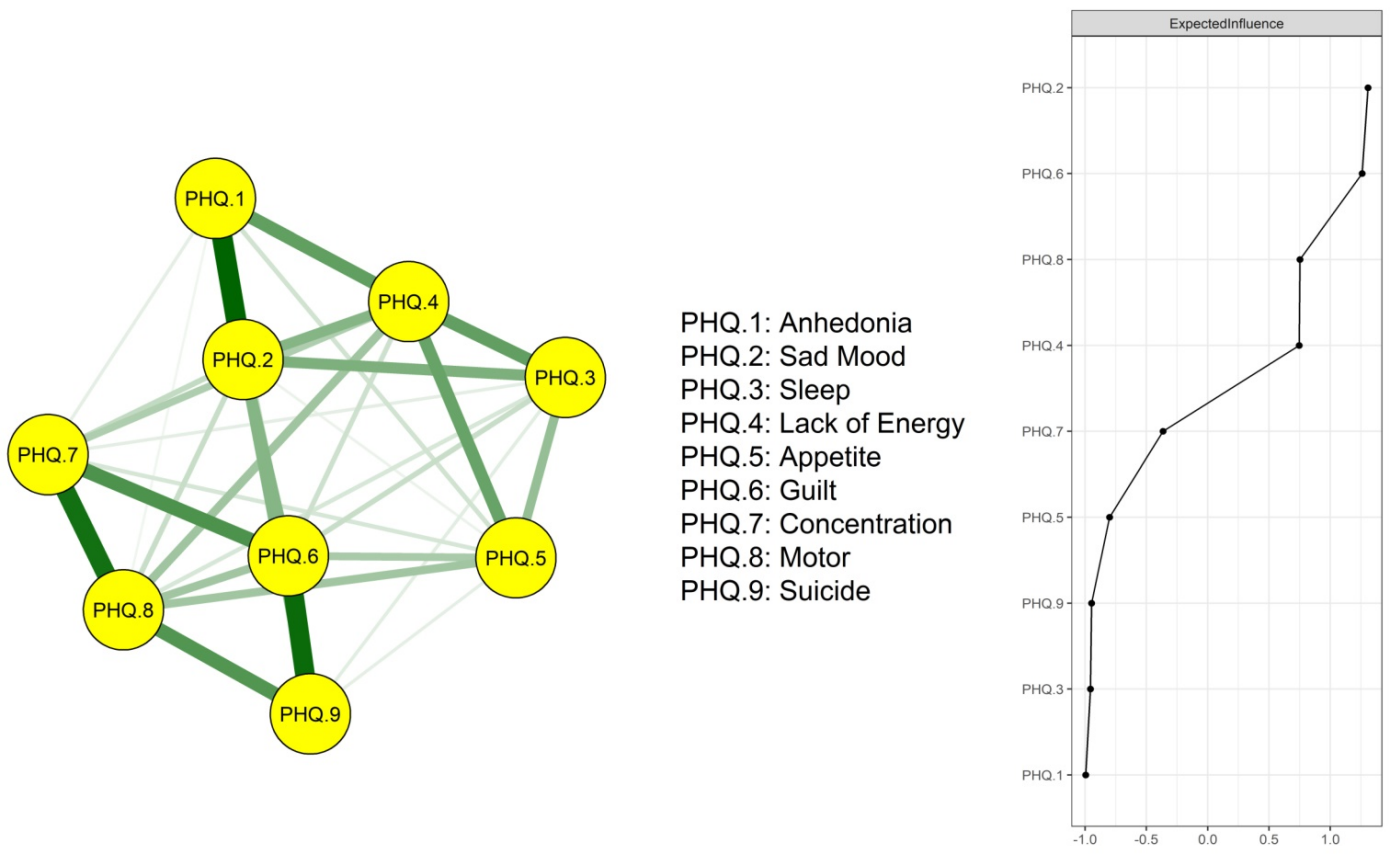
Estimated network model for dichotomized depressive symptoms and EI centrality in the whole sample (N = 3,946).

The network model of depressive symptoms was estimated (Figure 1; left panel). Strong positive correlations were found in Anhedonia-Sad Mood, Guilt-Suicide, Concentration-Motor, Guilt-Concentration, and Motor-Suicide. The numerical interactions between these symptoms were examined using a weighted adjacency matrix (Table S2). The right panel of Figure 1 shows the network's EI centrality. Symptom Sad Mood (item 2) and Guilt (item 6) showed the highest EI, followed by Motor problems (item 8) and Lack of Energy (item 4). Centrality measures including strength, betweenness and closeness are shown in Figure S1.

Strength and EI (CS-C=0.75) showed an excellent level of stability, indicating that when dropping approximately 75% of the observations, the strength index was still correlated at least 0.7 with the original network (Figure S2). The analysis of the accuracy of the edges, as implemented by means of non-parametric CIs, revealed that the precision of the edges was acceptable, with smaller CIs indicating more accurate estimations of the edges (Figure S3).

In terms of strength, Sad Mood (item 2) and Guilt (item 6) were the strongest, followed by Motor problems (item 8) and Lack of Energy (item 4) (Figure S4). Therefore, these four symptoms appeared to be particularly important and central to understanding depression among older adults in Hong Kong. Most of the comparisons were statistically significant among edge weights (Figure S5).

Descriptive statistics with frequencies of depressive symptoms by gender are shown in Table S3. Gender differences existed in the mean levels of depressive symptoms. Compared to males, females reported higher levels of Anhedonia, Sad Mood, Sleep problems, Lack of Energy, Appetite and Concentration (P <0.01).

**Figure 2:**
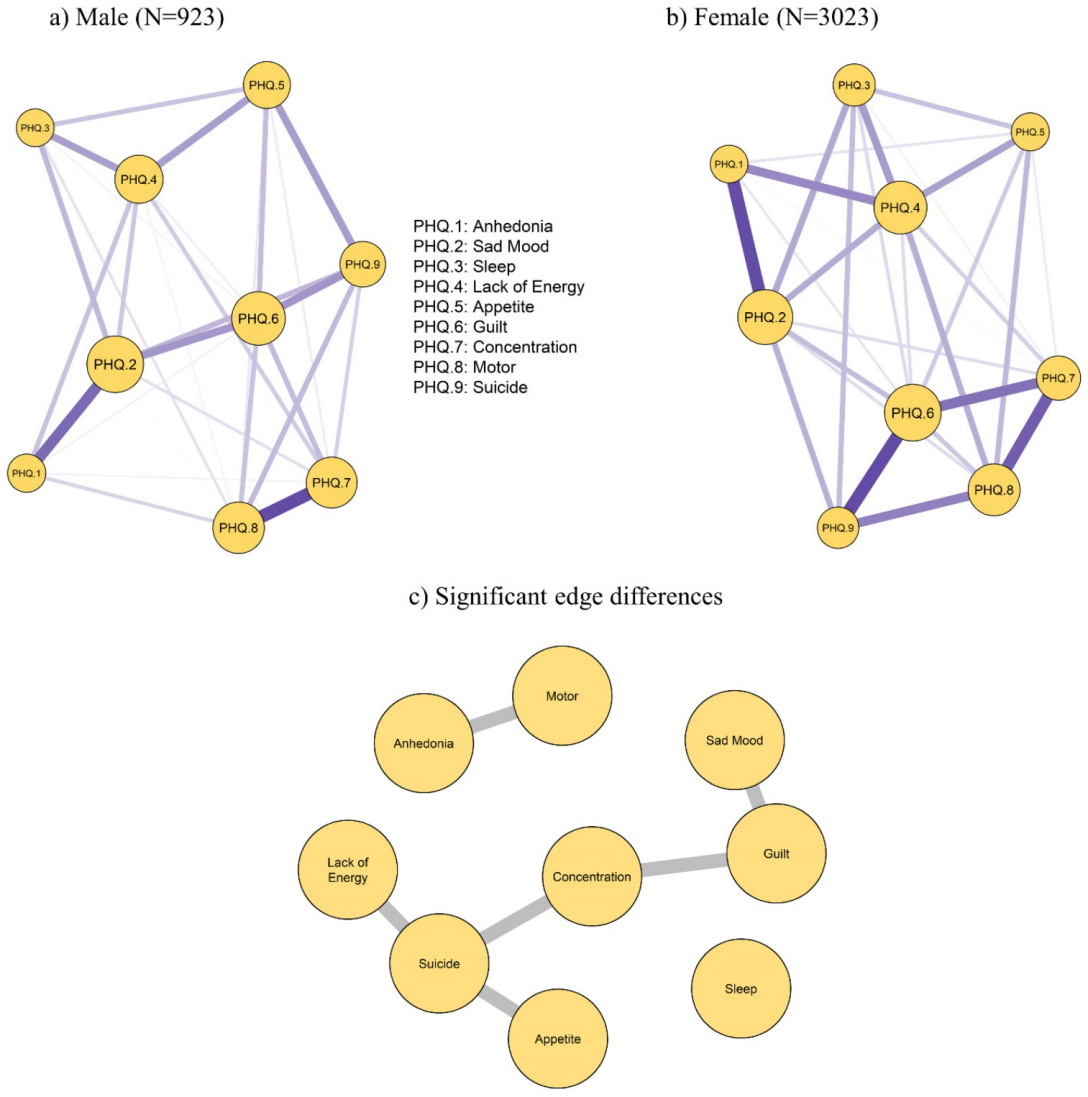
Estimated network models for dichotomized depressive symptoms in a) male (N = 923) and b) female (N = 3,023) participants; c) highlighting significant edge differences between the male and female sample (bottom panel).

The networks for the symptoms measured by PHQ-9 for men (left panel) and women (right panel) are presented in Figure 2. Although the threshold value is equal, the network structure for men is sparser than that for women. Centrality measures for PHQ-9 items by gender groups are displayed in Figure S6. The most central symptom in women was Guilt, followed by Sad Mood and Lack of Energy. While the most central symptom in men was Sad Mood, followed by Guilt and Motor problems.

Moreover, gender differences existed in network global strength: (global strength difference=0.14, p=0.004). After testing the statistical difference of each single edge between males and females using Bonferroni-Holm correction at p < 0.05 21, we found that the following edges between the two genders were significantly different: Sad Mood—Guilt, Concentration—Guilt, Anhedonia--Motor, Lack of Energy—Suicide, Appetite--Suicide and Concentration—Suicide (Table 2). The significant edge differences between both genders are displayed in Figure 2 (bottom panel).

## Discussion

The main finding of this study was that depressive symptoms were common in older adults in Hong Kong, particularly in women. In addition, Sad Mood, Guilt, Motor problems and Lack of energy were the central nodes in the network structure that might trigger or sustain the rest of the depressive symptoms among older adults during the pandemic. Moreover, significant gender differences were found in network global strength involving the following edges: Sad Mood--Guilt, Concentration--Guilt, Anhedonia--Motor, Lack of Energy--Suicide, Appetite--Suicide and Concentration--Suicide.

In a study using network analysis on MDD among older adults in Spain, depressed mood, sleep difficulties, guilt and loss of interest or pleasure were the core symptoms 25. In another study using network analysis among older adults in 19 European countries suicidal thoughts, sad mood, pessimism and guilt were the central symptoms of late-life depression 24. In this study, Sad Mood, Guilt, Motor problems and Lack of Energy were the central depressive symptoms in older adults in Hong Kong, which are partly consistent with previous findings 43-45, particularly concerning Sad Mood and Guilt. Sad Mood is related to feeling down, depressed or hopeless, which is considered to be one of the core symptoms of depression 46. In late life, sad mood may result from adverse life events (i.e., unemployment, bereavement and psychological trauma), chronic diseases, poor social support, and negative coping styles. In addition, the Hong Kong government had implemented various social distancing measures for the entire population, including mass quarantines, facility closures and restrictions on public transport 2, 47. All these measures might inevitably decrease access to treatments for health problems in older adults, which could subsequently increase their risk of having anxiety, social isolation, and disruptions to normal routines. Guilt was another central symptom in this study, which is similar to previous findings 48. The prevalence of chronic diseases among older people ranges from 60% to 82%, and nearly 50% of older people suffer from two or more chronic diseases 49. Chronic diseases may increase medical and financial burden on families which, in turn, may increase guilt feelings in older adults. The financial situation may be worse if any family member was made redundant or forced to stay at home without income during the pandemic. Financial strain and exposure to unsafe and unstable environments are commonly endorsed as stressful life events among older adults 50.

Motor problems and Lack of Energy were other central symptoms in older adults, both of which were not reported in previous studies 24, 25. The discrepancy could be due to differences in study periods (during vs. before the COVID-19 pandemic), measurements (PHQ-9 vs. other scales) and study sites (in China vs. Western countries). In this study, Motor problems refer to impairment in mobility or articulation/communication. Apart from declining physiological and cognitive functions 13, 51, older adults were often worried about the risk of infection and mortality caused by COVID-19. Older adults' concerns about higher mortality rates compared to other age groups 52 could affect their sleep quality and appetite 53, and subsequently, their energy levels. Moreover, restricted outdoor exercise due to quarantines during the pandemic could lead to a lack of energy in this subpopulation 54.

We found that the network structure of the depression model in men is sparser than in women, which is consistent with previous findings 25 and supports the notion of depressive symptoms in women as a more complex cluster of dynamic mechanisms compared to men 15. Furthermore, the most central symptoms in women included Guilt, followed by Sad Mood and Lack of Energy, while the most central symptoms in men were Sad Mood, followed by Guilt and Motor problems, suggesting central symptoms in both genders are almost similar. However, there were significant gender differences in network global strength, as older females have a stronger connection in the following edges: Sad Mood--Guilt, Concentration--Guilt, Anhedonia--Motor, Lack of Energy--Suicide, Appetite—Suicide, and Concentration--Suicide. Older males and females might show different network structures because of psychological, physiological, and socioeconomic factors. Most epidemiological studies have reported that psychological problems are more common in women than men 35, 36. Cognitive style often affects one's reactions to adverse events in life, and these reactions may differ based on the relationship between cognitive style and type of occurrence 55. This may explain the differences in depressive symptoms in older females compared with older males. Women may be psychologically more affected by predicaments than men, which can result in more prevalent and severe depressive symptoms in this population. For example, previous research suggests that there is a higher prevalence of social and health related concerns among females 2. Moreover, when facing stress and public health emergencies, women—who are often tasked with formal and informal caregiving roles and responsibilities—may be more exposed to these distressing situations compared to men 56-58.

Network analysis can provide an in-depth understanding of the characteristics of depressive symptoms among older adults. Central symptoms identified in the network model may serve as targets for treatments in depression, with interventions targeting the most prominent symptoms among older adults.

The strengths of this study included a large and homogenous study sample. State-of-the-art network analyses were adopted to ensure the trustworthiness and replicability of the results. However, several limitations need to be highlighted. First, due to the study's cross-sectional design, the causal relationships between symptoms examined could not be inferred. Second, older adults with acute physical diseases and those without internet access might have been excluded in a web-based survey, which can cause selection bias. Third, no function in the NCT could control for demographic and clinical variables that differ significantly between both genders; furthermore, physical health status and anxiety symptoms were not recorded in this study, both of which may influence the findings of the gender comparisons. Finally, data were collected based on self-reports, therefore we could not exclude the possibility that some participants might have undisclosed health conditions or medical history of depression, which may bias the results.

In conclusion, our results showed that Sad Mood, Guilt, Motor problems and Lack of Energy were the most central symptoms among older adults in Hong Kong during the pandemic. Additionally, significant gender differences existed in network global strength. Our findings provide a more nuanced understanding of depression and a framework for evaluating interventions that target depression in older adults. The increased association between Lack of Energy, Suicidal thoughts and Appetite among older females appears novel and may inform new theoretical considerations for gender differences in depression. Central symptoms identified in the network model among older adults may be prioritized in the treatment and prevention of depression during the pandemic.

## Supplementary Material

Supplementary figures and tables.

## Figures and Tables

**Table 1:** Socio-demographic characteristics of the study population (N= 3,946)

Variables	N	%
**Gender**		
Men	923	23.4
Women	3,023	76.6
Married/cohabiting	2,961	75.0
**Education level^ c^**		
Elementary or below ^a^	55	1.4
High school	998	25.3
College or higher	2,817	71.4
Low income ^b^	521	13.2
Living alone	162	4.1
	**Mean**	**SD**
Age (years)	56.63	5.73
PHQ-9 total score	6.03	5.47

Note: SD: standard deviation; PHQ-9: the 9-item Patient Health Questionnaire. ^a^ Elementary or below = less than 7 years of education. ^b^ Low income = low household's monthly income (< 100 hundred HKD 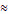 1.288 dollar). ^c^ There are missing values, therefore, the total percentage is not equal to 100%.

**Table 2:** Edge differences between male and female participants

Variable 1	Variable 2	P value
Anhedonia	Sad Mood	0.93
Anhedonia	Sleep	0.69
Sad Mood	Sleep	0.84
Anhedonia	Lack of Energy	0.10
Sad Mood	Lack of Energy	0.46
Sleep	Lack of Energy	0.47
Anhedonia	Appetite	0.18
Sad Mood	Appetite	0.82
Sleep	Appetite	0.83
Lack of Energy	Appetite	0.61
Anhedonia	Guilt	0.93
Sad Mood	Guilt	0.02*
Sleep	Guilt	0.75
Lack of Energy	Guilt	0.51
Appetite	Guilt	0.09
Anhedonia	Concentration	0.64
Sad Mood	Concentration	1.00
Sleep	Concentration	0.70
Lack of Energy	Concentration	0.69
Appetite	Concentration	0.89
Guilt	Concentration	0.03*
Anhedonia	Motor	0.04*
Sad Mood	Motor	0.30
Sleep	Motor	0.27
of Energy	Motor	0.50
Appetite	Motor	0.28
Guilt	Motor	0.94
Concentration	Motor	0.10
Anhedonia	Suicide	1.00
Sad Mood	Suicide	0.51
Sleep	Suicide	0.17
Lack of Energy	Suicide	0.03*
Appetite	Suicide	<0.001*
Guilt	Suicide	0.15
Concentration	Suicide	0.01*
Motor	Suicide	0.62

Note: Bolded values: <0.05; *significant after Bonferroni correction.

## Abbreviations

COVID-19: The 2019 novel coronavirus disease
SARS: severe acute respiratory syndrome
PHQ-9: Patient Health Questionnaire-9
DSM-IV: Diagnostic and Statistical Manual of Mental Disorders, Fourth Editions
MDD: major depressive disorder
SD: standard deviation
eLASSO: enhanced least absolute shrinkage and selection operator
EBIC: extended Bayesian Information Criterion
EI: excepted influence
CIs: confidence intervals
CS-C: correlation stability coefficient
NCT: Network Comparison Test
