# Supplementary material for: Depressive symptoms and gender differences in older adults in Hong Kong during the COVID-19 pandemic: a network analysis approach
Source: Int J Biol Sci. 2022 Jun 6;18(10):3934–41. doi: 10.7150/ijbs.69460 (PMC9274487; doi:10.7150/ijbs.69460)

Supplementary materials

Supplementary Information

STable 1. Mean, standard deviation, minimum, maximum, skewness, kurtosis, and frequency of depressive symptoms as measured by the PHQ-9 (n = 3,946).

STable 2. Weighted adjacency matrix.

STable 3. Mean, Standard Deviation, Skewness, Kurtosis, and Frequency of depressive Symptoms as measured using the PHQ-9 by gender.

SFigure 1. Centrality measures of all symptoms within the network.

SFigure 2. Stability of centrality indices by case dropping subset bootstrap.

SFigure 3. Bootstrapped confidence intervals of edge weights.

SFigure 4. Estimations of node strength difference by bootstrapped difference test.

SFigure 5. Estimations of edge weight difference by bootstrapped difference test.

SFigure 6. Comparisons of network centrality indices between male and female participants.

STable 1. Mean, standard deviation, minimum, maximum, skewness, kurtosis, and frequency of depressive symptoms as measured by the PHQ-9 (n = 3,946).

Depressive symptoms	PHQ-9 item	M	SD	Min	Max	Skewness	Kurtosis	% (Absence)	% (Presence)
Anhedonia	1	0.61	0.49	0	1	-0.46	-1.79	38.8	61.2
Sad Mood	2	0.56	0.50	0	1	-0.25	-1.94	43.9	56.1
Sleep	3	0.62	0.49	0	1	-0.48	-1.77	38.4	61.6
Lack of Energy	4	0.61	0.49	0	1	-0.43	-1.81	39.4	60.6
Appetite	5	0.51	0.50	0	1	-0.03	-2.00	49.2	50.8
Guilt	6	0.33	0.47	0	1	0.74	-1.45	67.4	32.6
Concentration	7	0.44	0.50	0	1	0.25	-1.94	56.1	43.9
Motor	8	0.28	0.45	0	1	0.99	-1.01	72.3	27.7
Suicide	9	0.08	0.28	0	1	2.98	6.90	91.5	8.5

Note: M: Mean; Min: Minimum; Max: Maximum; PHQ-9: The Patient Health Questionnaire-9; SD: Standard Deviation.

STable 2. Weighted adjacency matrix

Depressive symptoms	Anhedonia	Sad Mood	Sleep	Lack of Energy	Appetite	Guilt	Concentration	Motor	Suicide
Anhedonia	0.00	1.90	0.14	1.21	0.43	0.45	0.29	0.20	0.00
Sad Mood	**1.90**	0.00	1.02	0.95	0.24	0.95	0.52	0.48	0.95
Sleep	0.14	1.02	0.00	1.21	0.84	0.48	0.29	0.38	0.30
Lack of Energy	1.21	0.95	1.21	0.00	1.16	0.44	0.67	0.77	0.00
Appetite	0.43	0.24	0.84	1.16	0.00	0.71	0.39	0.76	0.28
Guilt	0.45	0.95	0.48	0.44	0.71	0.00	1.35	0.76	1.82
Concentration	0.29	0.52	0.29	0.67	0.39	**1.35**	0.00	1.76	0.00
Motor	0.20	0.48	0.38	0.77	0.76	0.76	**1.76**	0.00	1.32
Suicide	0.00	0.95	0.30	0.00	0.28	**1.82**	0.00	**1.32**	0.00

Weighted adjacency matrix based on factors to represent the weight of direct edges between nodes.

SFigure 1. Centrality measures of all symptoms within the network. The figure shows centrality measure (i.e., strength, betweenness, and closeness) of all symptoms within the network (z-scores).

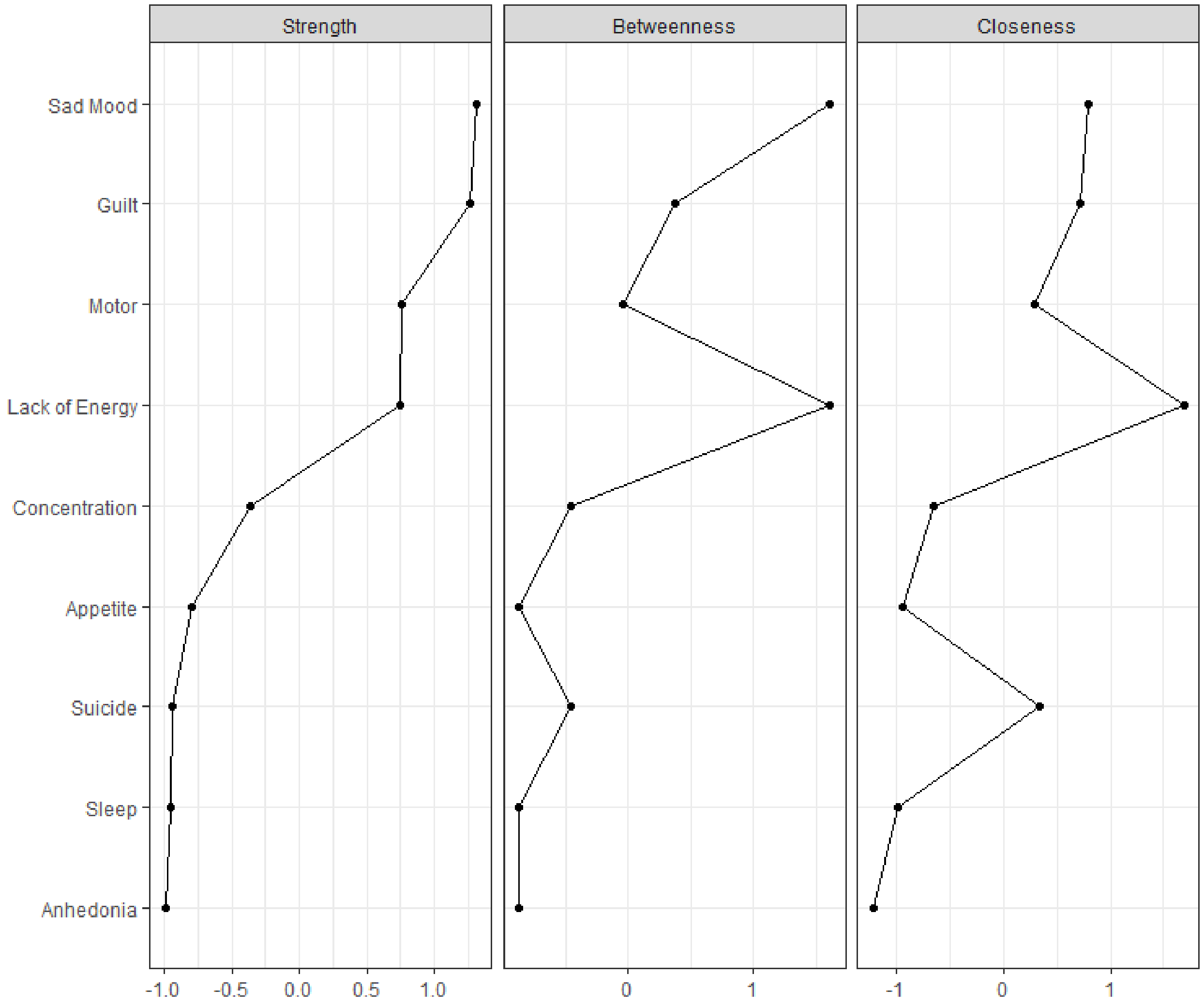

SFigure 2. Stability of centrality indices by case dropping subset bootstrap. The x-axis represents the percentage of cases of the original sample used at each step. The y-axis represents the average of correlations between the centrality indices in the original network and the centrality indices from the re-estimated networks after excluding increasing percentages of cases. Each line indicates the correlations of betweenness, closeness, EI (expected influence) and strength.

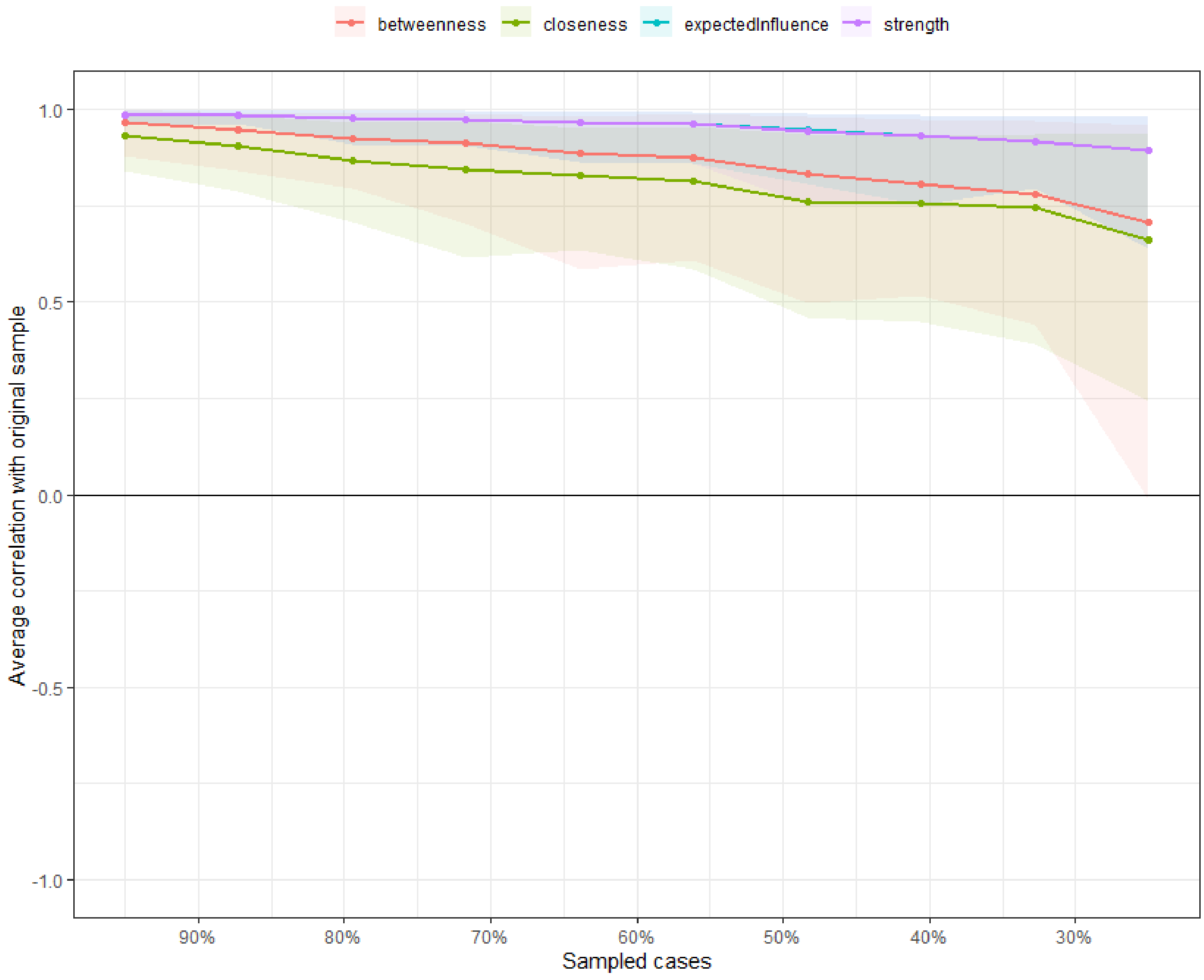

SFigure 3. Bootstrapped confidence intervals of edge weights. The black dots indicate the values of each edge weight, ordered from the highest to the lowest value. The gray area represents the 95% Confidence Intervals of edge weights, estimated with the non-parametric bootstrap procedure (Bootnet package). Wide intervals indicate lower stability and narrow intervals indicate higher stability.

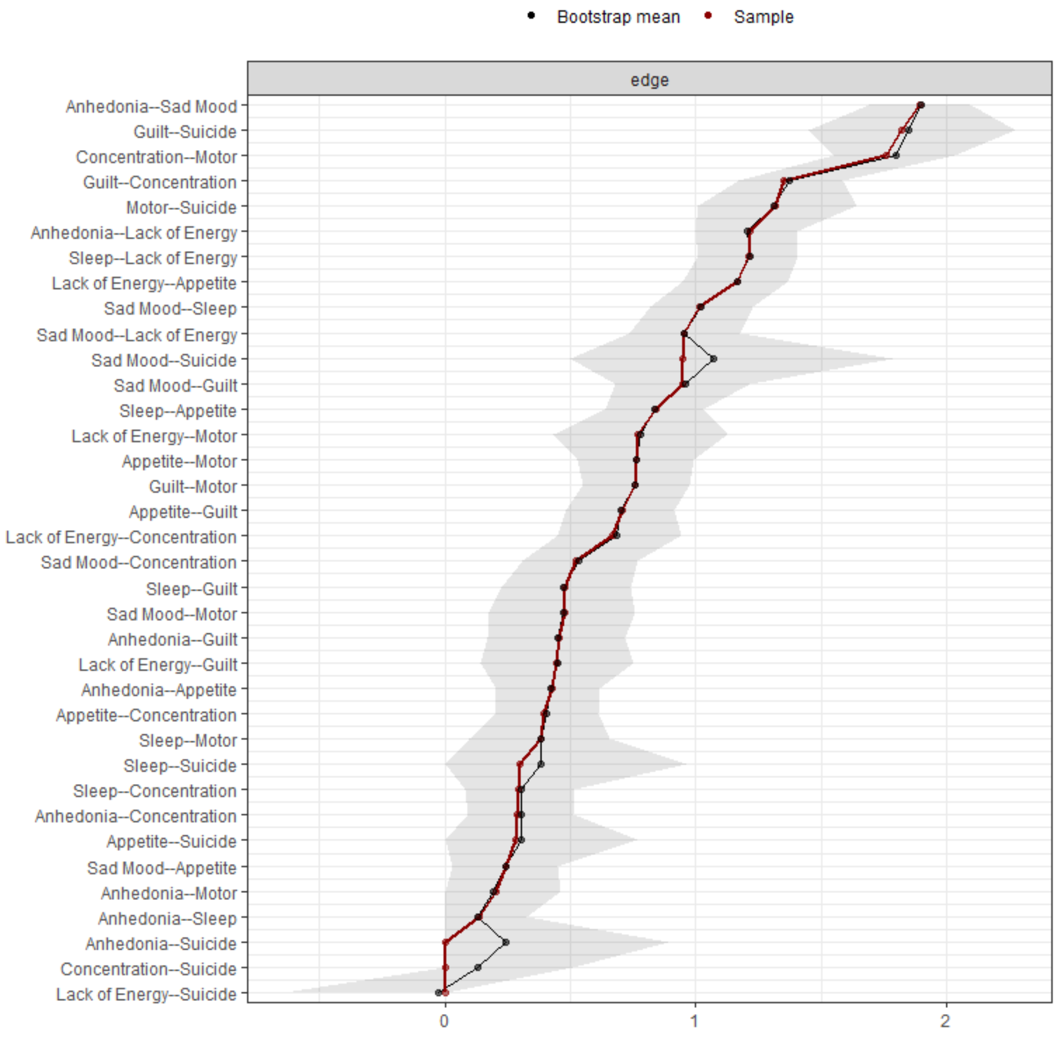

SFigure 4. Estimation of node strength difference by bootstrapped difference test. Bootstrapped difference tests between node strength of individual symptoms. Gray boxes indicate nodes that do not significantly differ from one-another. Black boxes represent nodes that differ significantly from one another (α = 0.05). White boxes show the values of node strength.

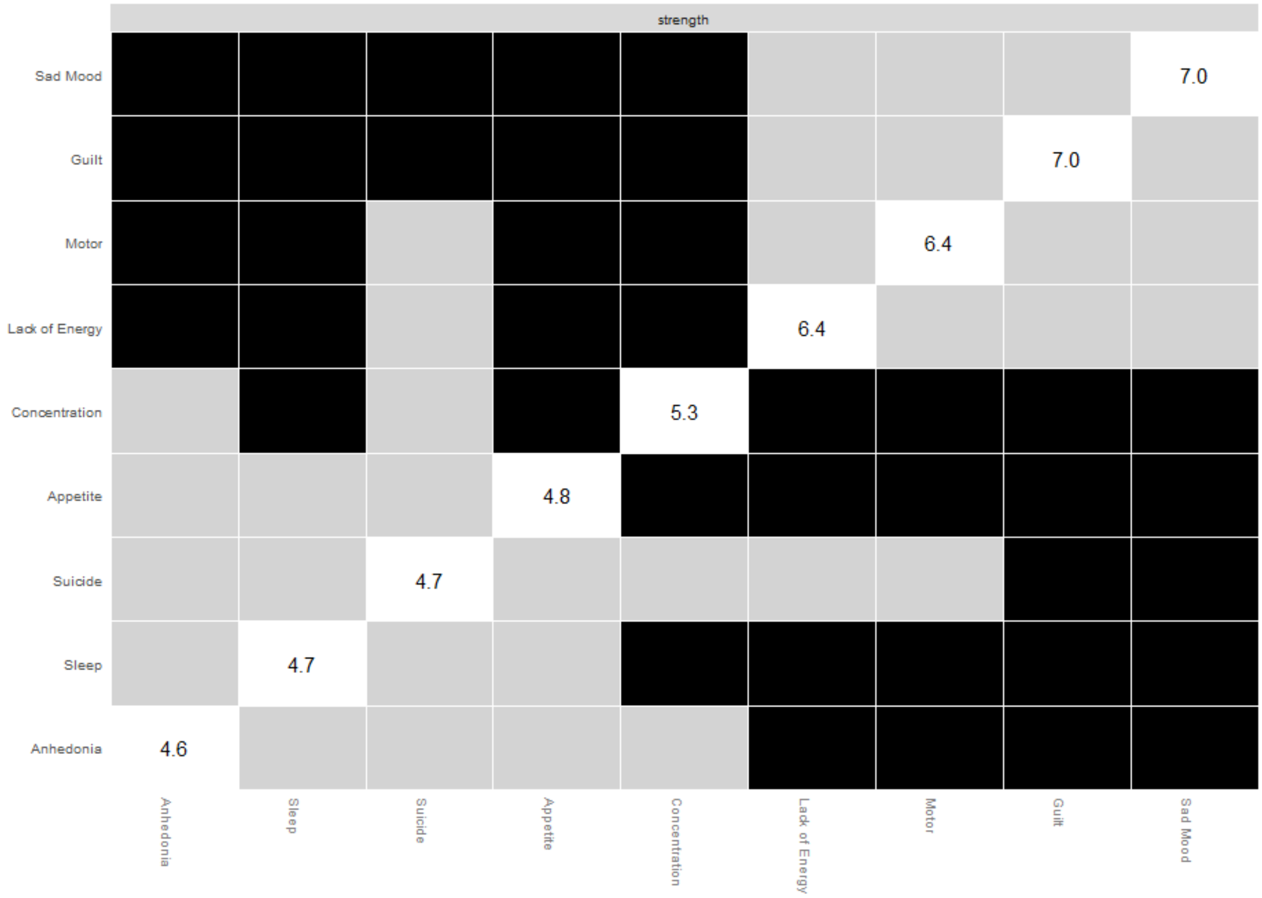

SFigure 5. Estimation of edge weight difference by bootstrapped difference test. Bootstrapped difference tests between edge weights in the network. Gray boxes indicate edges that do not significantly differ from one-another. Black boxes represent edges with significant difference from one another (α = 0.05). Blue boxes in the edge-weight plot indicate positive correlations.

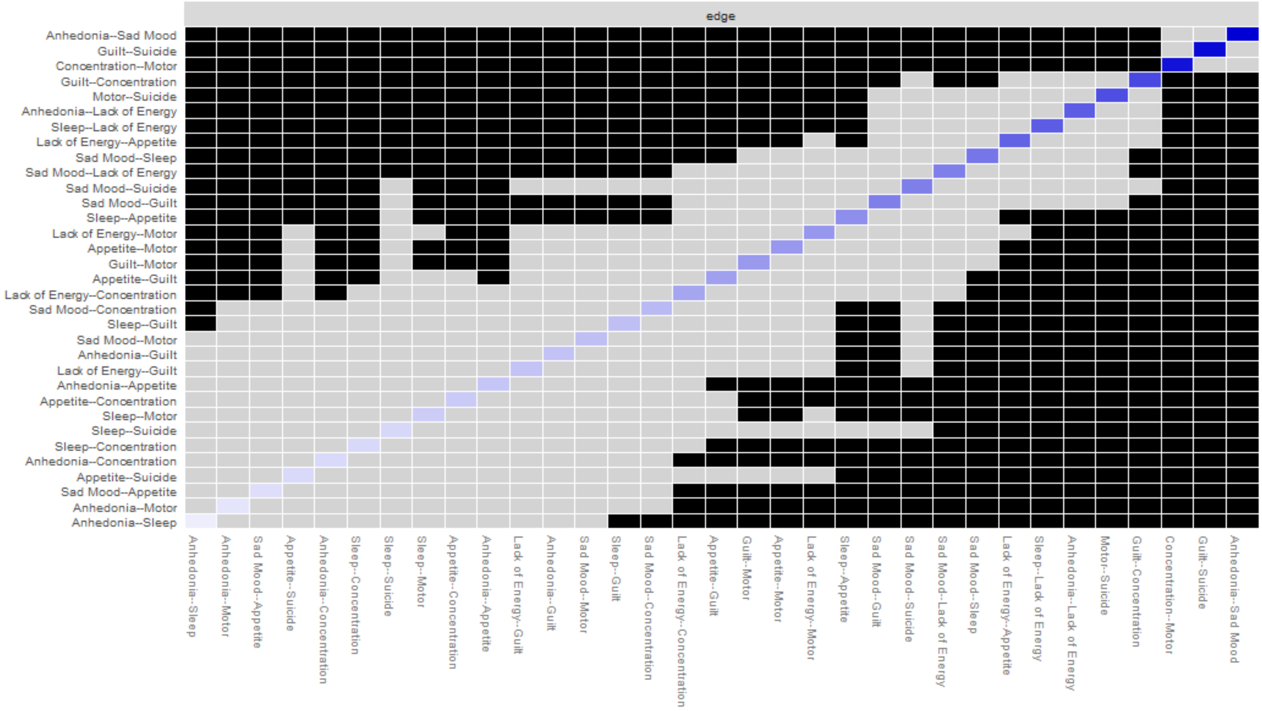

STable 3. Mean, Standard Deviation, Skewness, Kurtosis, and Frequency of depressive symptoms as measured using the PHQ-9 by gender.

	Male (N=923)						Female (N=3,023)							
Depressive symptoms	M	SD	Skewness	Kurtosis	% Absence	% Presence	M	SD	Skewness	Kurtosis	% Absence	% Presence	t-test	P
Anhedonia	0.56	0.50	-0.25	-1.94	43.9	56.1	0.63	0.48	-0.53	-1.72	37.2	62.8	3.64	**<0.01**
Sad Mood	0.49	0.50	0.04	-2.00	50.9	49.1	0.58	0.49	-0.33	-1.89	41.8	58.2	4.91	**<0.01**
Sleep	0.51	0.50	-0.04	-2.00	49.1	50.9	0.65	0.48	-0.62	-1.61	35.2	64.8	7.67	**<0.01**
Lack of Energy	0.50	0.50	-0.02	-2.00	49.6	50.4	0.64	0.48	-0.57	-1.68	36.3	63.7	7.30	**<0.01**
Appetite	0.40	0.49	0.41	-1.83	60.1	39.9	0.54	0.50	-0.17	-1.97	45.8	54.2	7.67	**<0.01**
Guilt	0.32	0.47	0.75	-1.44	67.6	32.4	0.33	0.47	0.74	-1.45	67.4	32.6	0.14	0.88
Concentration	0.38	0.49	0.50	-1.75	62.2	37.8	0.46	0.50	0.17	-1.97	54.3	45.7	4.24	**<0.01**
Motor	0.25	0.43	1.17	-0.63	75.3	24.7	0.29	0.45	0.94	-1.11	71.3	28.7	2.36	0.02
Suicide	0.09	0.29	2.86	6.20	91.0	9.0	0.08	0.28	3.02	7.13	91.7	8.3	0.66	0.51

SFigure 6. Comparisons of network centrality indices between male and female participants.

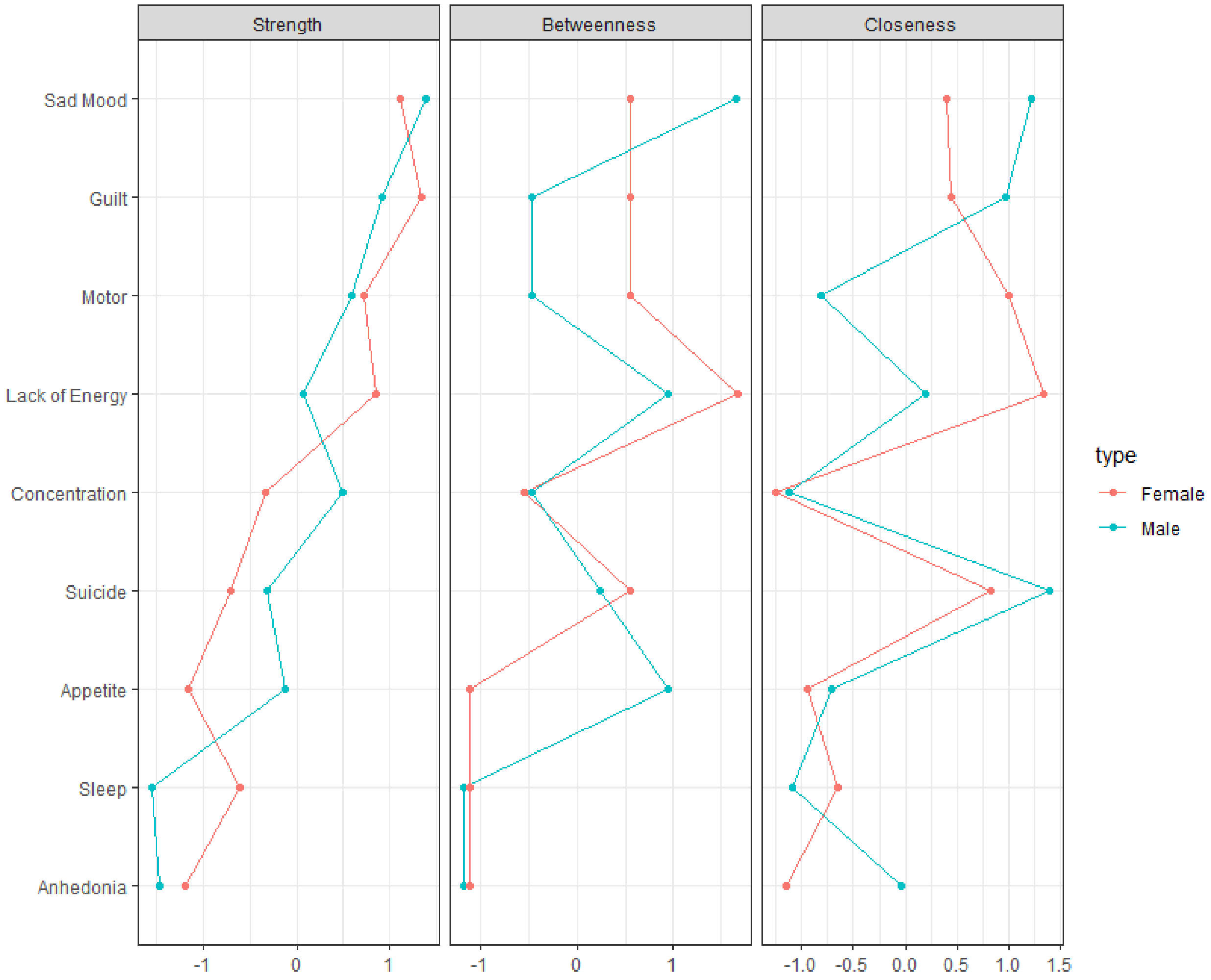

Supplement: Supplementary file 1 — Supplementary figures and tables. [file ijbsv18p3934s1.pdf]
